# Novel machine‐learning bioinformatics reveal distinct metabolic alterations for enhanced colorectal cancer diagnosis and monitoring

**DOI:** 10.1002/imo2.70003

**Published:** 2025-03-03

**Authors:** Rui Xu, Hyein Jung, Fouad Choueiry, Shiqi Zhang, Rachel Pearlman, Heather Hampel, Ning Jin, Jieli Li, Jiangjiang Zhu

**Affiliations:** ^1^ Human Nutrition Program, Department of Human Sciences The Ohio State University Columbus Ohio USA; ^2^ Comprehensive Cancer Center The Ohio State University Columbus Ohio USA; ^3^ Department of Chemistry and Biochemistry The Ohio State University Columbus Ohio USA; ^4^ Department of Internal Medicine The Ohio State University Columbus Ohio USA; ^5^ Department of Medical Oncology & Therapeutics Research City of Hope National Cancer Center Duarte Ohio USA; ^6^ Division of Clinical Cancer Genomics, Beckman Research Institute City of Hope California USA; ^7^ Department of Pathology The Ohio State University Columbus Ohio USA

**Keywords:** artificial neural network, colorectal cancer, metabolomics, multi‐omics, partial least squares discriminant analysis, transcriptomics

## Abstract

Colorectal cancer (CRC) is the second leading cause of cancer‐related mortality in the United States when considering both men and women. Colonoscopy remains the gold standard for CRC diagnosis but is invasive, costly, and requires extensive bowel preparation and sedation. Recent advancements in high throughput “omics” technologies may offer less invasive methods for CRC diagnosis through biomarker discovery. This study introduces a novel bioinformatics pipeline, PLS‐ANN‐DA (PANDA), combining partial least squares discriminant analysis (PLS‐DA) with an advanced artificial neural network (ANN) to improve CRC diagnosis and monitor disease progression. We analyzed metabolic alterations in CRC using a metabolomics data set of 626 CRC cases and 402 healthy controls (HC). Meanwhile, complementary transcriptomic data were also analyzed and integrated to further understand CRC metabolic dysregulations. By integrating metabolomics and transcriptomics analyses and establishing the biomarker discovery pipeline PANDA, significant metabolic pathway alterations were identified between CRC patients and healthy controls, with notable upregulation of multiple pathways in CRC. Meanwhile, we observed a downregulation of specific pathways, including purine metabolism and the tricarboxylic acid (TCA) cycle, associated with advanced tumor stages. The PANDA pipeline showed promising outcomes by effectively differentiating CRC from healthy states and providing insight into metabolic shifts occurring in advanced CRC stages. Genetic mutation‐associated metabolic changes were also discovered. Overall, this method has the potential for noninvasive CRC diagnostics and may serve as a valuable tool for understanding metabolic changes in cancer progression.

## Highlights

- Significant upregulation of key metabolic pathways, including the tricarboxylic acid (TCA) cycle, purine metabolism, and amino acid metabolism, was observed in colorectal cancer (CRC) cases compared to healthy controls.
- Metabolic shifts correlated with tumor (T) and lymph node (N) stages, including increased pyruvic acid levels and decreased phenol levels in metastatic cases.
- Purine metabolism showed distinct patterns, with upregulation in CRC but downregulation in advanced tumor stages, linked to oncogenic signaling and nutrient deprivation.
- The PANDA pipeline effectively integrated metabolomic and transcriptomic data, enhancing robustness in CRC biomarker discovery and progression monitoring.

## Graphical abstract

The PANDA pipeline for colorectal cancer (CRC) analysis integrates metabolomic data from LC‐MS with machine learning techniques to classify CRC stages and predict biomarkers. Initially, the raw metabolomic data is processed using partial least squares discriminant analysis (PLS‐DA) to reduce dimensionality and highlight key features. These selected features are then used to train a neural network, which learns to classify CRC stages (T1–T4) and predict relevant biomarkers. This approach allows for a more accurate diagnosis, early detection, and identification of potential therapeutic targets, contributing to personalized treatment strategies for CRC patients.

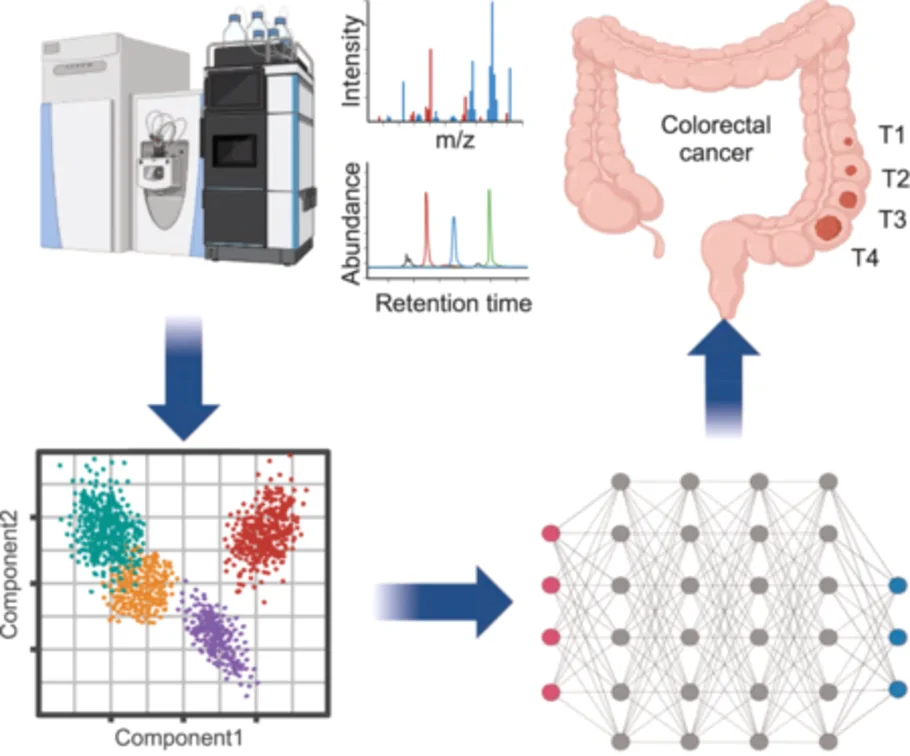

## 1 INTRODUCTION

Colorectal cancer (CRC) stands as the third most frequently diagnosed cancer and ranks third among the leading causes of cancer‐related mortality in both males and females within the United States [1]. Projections indicate a substantial surge of 60% in its prevalence, anticipating more than 2.2 million new CRC cases and 1.1 million cancer‐related fatalities by the year 2030 [2]. A large proportion of individuals diagnosed with stage I and II colon cancer typically undergo colectomy [3] as the primary treatment modality, with a notable majority (84%) not recommended to pursue chemotherapy [4, 5], because stage I colon and rectal cancers, the 5‐year survival rate reaches to 95% and the recurrence rate is as low as 5%. However, around two‐thirds of patients with stage III colon cancer and about 90% of patients with stage IV colon cancer undergo adjuvant chemotherapy [4], with the 5‐year survival at 45%–70% and a recurrence rate of 33%. Thus, ensuring widespread access to high‐quality screening and treatment across diverse populations holds the potential to expedite advancements in combatting this prevalent cancer.

The majority of diagnoses typically occur in individuals aged 65 and older, while 13% of cases are observed in individuals under the age of 50, with an additional 33% occurring in the 50–64 age group [6]. Despite the ongoing increase in CRC cases overall, there is a notable trend towards diagnosis at earlier ages, with progression to more severe stages [7]. Thus, new recommendations advise individuals aged 45–49 to get screened for early‐onset disease via stool tests or structural examinations (colonoscopy), enhancing chances of treatment response [1, 8, 9]. According to a 2021 report, just 67% of individuals in the United States have undergone CRC screening to date [10]. While colonoscopy is effective in detecting CRC and removing polyps, it's invasive, costly, and demands significant resources [11]. Hence, there is an urgent clinical demand for alternative screening methods of less invasive, easily accessible, and more convenient testing [12]. In addition, CRC development is multifactorial, with genetic mutations being pivotal alongside lifestyle influences. Cancer cells acquire the effective reprogramming of cell metabolism, enabling them to meet increased nutrient and energy demands [13]. Thus, advanced high throughput “omics” methodologies such as metagenomics, transcriptomics, proteomics, and metabolomics present a potentially less invasive avenue for diagnosing CRC [14, 15]. Each of these techniques offers unique advantages in cancer biomarker discovery and diagnosis. Alongside advancements in high‐throughput technologies, data analysis methods have also evolved, making bioinformatics an invaluable tool for enhancing our understanding and prediction of CRC. By integrating multi‐omics data, bioinformatics provides a robust framework to analyze various molecular layers, offering a comprehensive view of CRC's pathological mechanisms [16]. Combined with artificial intelligence and machine learning, bioinformatics not only improves the efficiency of data integration but also enhances our ability to recognize complex patterns in cancer progression. These approaches support molecular subtyping and biomarker discovery, contributing to more precise assessments of patient prognosis and treatment responses [14]. Within this context, bioinformatics has become essential for unraveling cancer's complex molecular mechanisms, improving early diagnosis, and tracking disease progression to ultimately enhance patient outcomes.

In this study, we utilized metabolomics data, validated and integrated it with publicly accessible transcriptomic data to study the metabolic abnormalities of CRC patients at different cancer stages. Concurrently, we have designed a new data analysis pipeline integrating the methodologies of both partial least squares discriminant analysis (PLS‐DA) and advanced artificial neural network (ANN), specifically, feedforward networks, named PLS‐ANN‐DA (PANDA), to enhance CRC diagnosis and progression monitoring. PLS‐DA is suitable for scenarios involving small sample sizes or high‐dimensional feature data, while ANN excels in handling large‐scale and complex pattern recognition tasks. Therefore, in this study, where both high sample sizes and high‐dimensional features are present, PLS is utilized to extract the most discriminative features and reduce data dimensionality. Subsequently, ANN leverages these features to construct more complex models, enhancing the capture of complex patterns and associations within the data, thereby improving model accuracy and robustness in DA. Our aim is to develop a new bioinformatic pipeline through our extensive omics data to have a comprehensive understanding of the metabolic dysregulations in CRC pathology and propose promising molecular biomarkers of CRC diagnosis and progression for future translations into clinical applications.

## 2 RESULTS

### 2.1 The advanced pipelines to study changes in metabolic profiles for CRC diagnosis and progression

**FIGURE 1:**
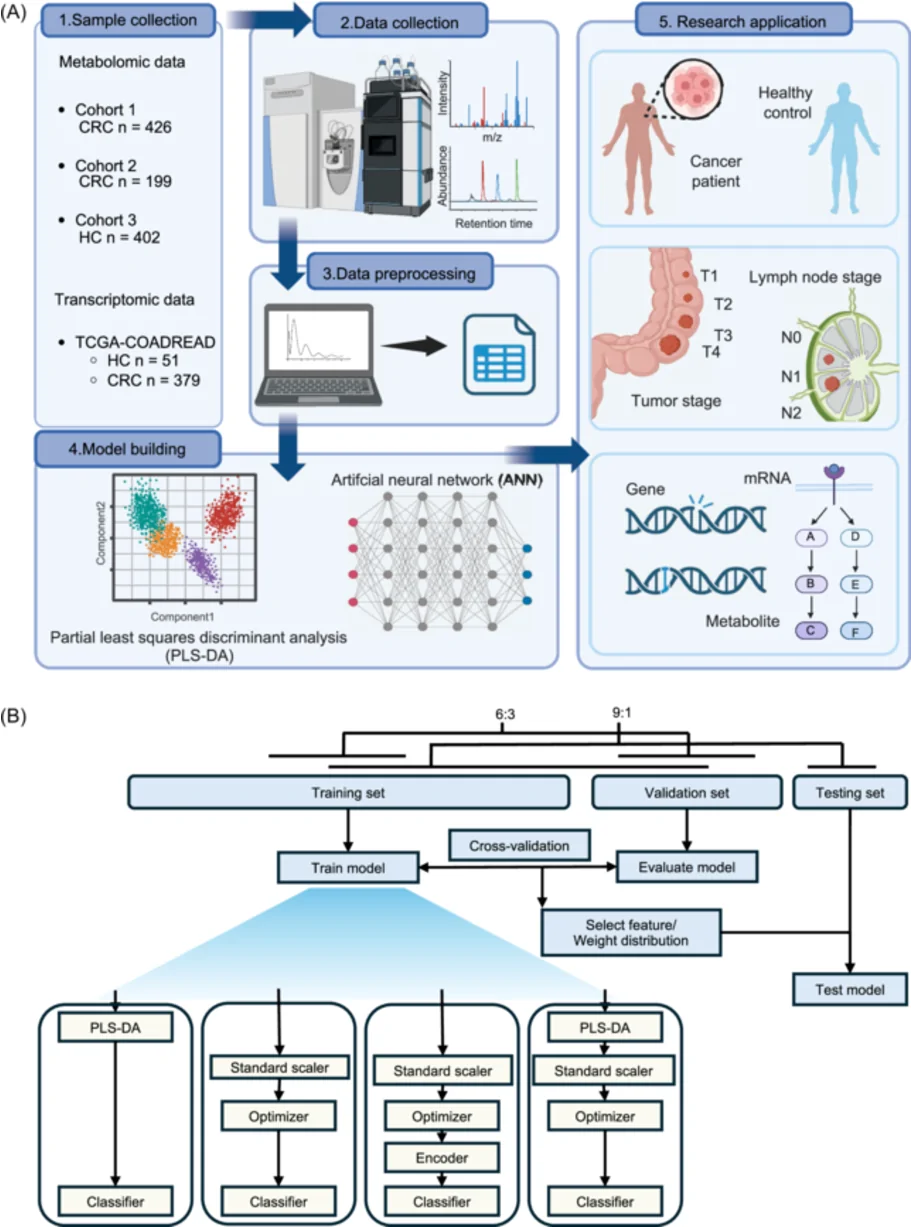
Workflow of integrative analysis of metabolomic and transcriptomic data in colorectal cancer (CRC), including cross‐validation and feature selection. (A) The schematic workflow of integrative analysis of metabolomic and transcriptomic data in CRC. (B) Cross‐validation and four different feature selection and classification workflow.

The schematic workflow depicted in Figure 1 delineates the methodological framework and statistical model employed in this study. Three distinct cohorts of samples were obtained for metabolomics analyses, while an additional cohort of transcriptomic data was acquired from TCGA databases to serve as validation of findings from metabolomics results and facilitate multi‐omics integration analyses (Figure 1A). Following metabolomic data collection, a rigorous preprocessing protocol was implemented. Overall, 240 metabolites were confidently annotated via standard references and high‐resolution databases. Subsequently, both PLS‐DA and ANN models were deployed to address key research inquiries, encompassing the identification of diagnostic and prognostic biomarkers, as well as delineating mutation‐associated dysfunctional pathways. In Figure 1B, we demonstrate the application of benchmark classification methods, including the separate use of PLS‐DA and ANN, and the development of the advanced in‐tandem pipeline of these methods, PANDA, in model classification. Simultaneously, we utilize cross‐validation to ensure the reliability and generalizability of our classification results. For each analysis, the samples underwent a division into a 90:10 ratio for model training and testing. Within the model training set, a subsequent split was made into a 60:30 ratio for training and validation purposes in each loop of cross‐validation. Training was conducted for 100 epochs. Four workflows were performed for model training. The PLS‐DA workflow entails the construction of a PLS‐DA plot using the training set and validation set, thereby establishing a 95% confidence interval area. Then, a PLS‐DA classifier was adopted, wherein the Mahalanobis distance of the additional testing set is computed, aiming to inform the classification decision by providing a measure of the dissimilarity between data points. The subsequent three workflows utilize ANN classifiers, incorporating z‐score normalization and hyperparameter optimization via standard scaler and optimizer techniques. Hyperparameters were optimized for parameters including hidden layers, layer size, learning rate, activation, regularization, regularization strength, and batch size. After identifying the optimal parameters, three different feature sets were input into the ANN classifier, including raw features, features after autoencoding, and 8 components from the corresponding PLS‐DA model. In contrast to the second workflow, the third workflow condenses all normalized features into a 2–10‐dimensional space, selecting the most optimal representative reduction for subsequent classification. The fourth and final workflow is our innovative PANDA pipeline, which amalgamates all features into 8 components using PLS, followed by an intricate mapping function through ANN, culminating in discriminative analysis conducted by the classifier.

### 2.2 Comparative metabolic profiling of CRC patients against HC

**FIGURE 2:**
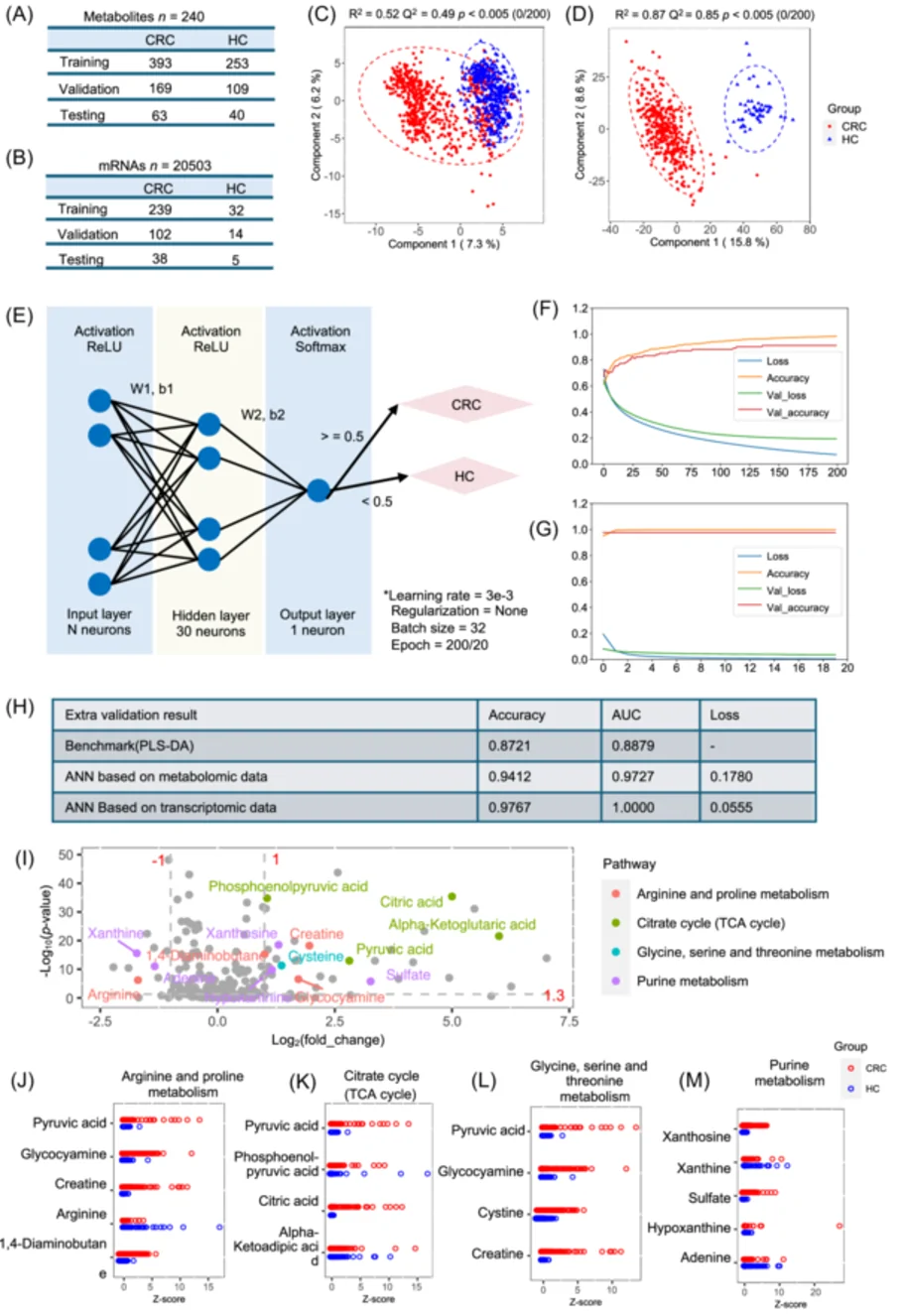
Comparison of colorectal cancer (CRC) versus healthy controls (HC) datasets, partial least squares discriminant analysis (PLS‐DA) and artificial neural network (ANN) model training, evaluation, and Z‐Score analysis of dysregulated metabolites in various pathways. (A) Data set sizes for CRC versus HC analysis ‐ metabolomic data comparison. (B) Data set sizes for CRC versus HC analysis ‐ transcriptomic data comparison. (C) PLS‐DA model training and validation ‐ metabolomic data. (D) PLS‐DA model training and validation ‐ transcriptomic data. (E) ANN model optimization for CRC versus HC classification. (F) ANN model training ‐ metabolomic data. (G) ANN model training ‐ transcriptomic data. (H) Model evaluation ‐ testing data set performance. (I) Volcano plot analysis of dysregulated metabolites in CRC pathways. (J) Z‐Score analysis of dysregulated metabolites in arginine and proline metabolism pathway. (K) Z‐Score analysis of dysregulated metabolites in citrate cycle (TCA cycle) pathway. (L) Z‐Score analysis of dysregulated metabolites in glycine, serine, and threonine metabolism pathway. (M) Z‐Score analysis of dysregulated metabolites in purine metabolism pathway.

To discover the metabolic disparities between CRC and HC cohorts, we employed both PLS‐DA and ANN modeling techniques on plasma metabolite data. To corroborate the discriminative capacity of the metabolite data set between the two groups, we also performed transcriptomics analyses of TCGA data. Figure 2A,B presents the data set sizes for the CRC versus HC analysis utilizing both metabolomics data and TCGA transcriptomic data. Subsequently, employing training and validation sets, PLS‐DA models were constructed for each type of data, as illustrated in Figure 2C,D. Despite the transcriptomic data demonstrating superior separation between CRC and HC, both models exhibited commendable fitting ability (*R* ^2^ = 0.52 and 0.87) and predictive performance (*Q* ^2^ = 0.49 and 0.85). Notably, despite that the mRNA data size is much larger compared to the sample size in the transcriptomic data set, neither model exhibited signs of overfitting, as confirmed by permutation tests (*p* < 0.005). To enhance the classification of CRC and HC, ANN model was also applied to both datasets. Hyperparameter optimization led to the introduction of a single hidden layer comprising 30 neurons, alongside other parameters detailed in Figure 2E. Following 200 epochs and 20 epochs of training for metabolomic and transcriptomic data, respectively, the loss function reduced to below 0.2 in both the training and validation datasets, with accuracy exceeding 0.95 (Figure 2F,G). Subsequently, the testing data set was utilized to assess the aforementioned models, with accuracy, area under the curve (AUC), and loss reported in Figure 2H. It was observed that both the PLS‐DA (accuracy = 0.8721, AUC = 0.8879) and the ANN‐based model (accuracy = 0.9412, AUC = 0.9727) have a good separation prediction between HC and CRC cases. Meanwhile, in ANN models, both the metabolomic data (accuracy = 0.9412, AUC = 0.9727, loss = 0.1780) and the transcriptomic data (accuracy = 0.9767, AUC = 1, loss = 0.0555) exhibited superior performance on the classification.

Meanwhile, the volcano plot analysis was employed to identify significantly altered metabolites in CRC compared to HC. As illustrated in Figure 2I, notable metabolites within the four primary dysregulated pathways, namely arginine and proline metabolism, citrate cycle (TCA cycle), glycine, serine, and threonine metabolism, and purine metabolism, were highlighted. In the context of CRC, a majority of the identified metabolites exhibited a statistically significant increase in abundance, with the exception of xanthine, adenine, and arginine. Furthermore, to comprehensively assess the dysregulation within these pathways, z‐score was computed for the identified metabolites. As depicted in Figure 2J–M, the observed trends predominantly indicate upregulation within these pathways in CRC cases.

### 2.3 Metabolic alterations across T and N stages in CRC

**FIGURE 3:**
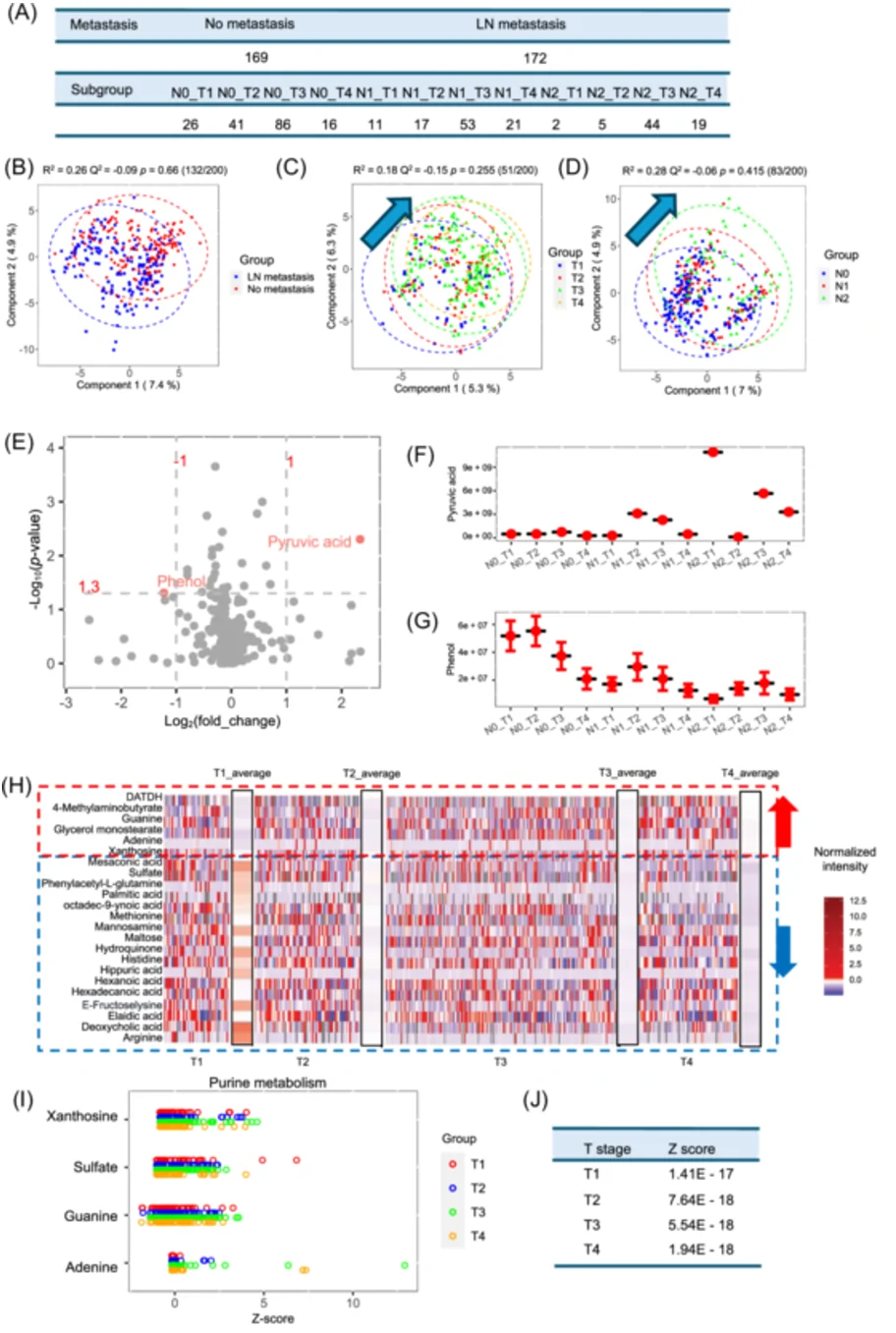
Stratification of colorectal cancer (CRC) subsets by tumor and lymph node stages, partial least squares‐discriminant analysis (PLS‐DA) model comparisons, and analysis of altered compounds and dysregulated metabolites across stages. (A) CRC subset with detailed staging information stratification by tumor and lymph node (LN) stages. (B) PLS‐DA model ‐ with and without metastasis groups. (C) PLS‐DA model ‐ tumor stage comparison. (D) PLS‐DA model ‐ lymph node stage comparison. (E) Volcano plot analysis of metabolites in with and without metastasis groups. (F) Compound analysis by lymph node (LN) stage. (G) Compound analysis by tumor (T) stage. (H) Selected altered compounds by tumor and lymph node (LN) stages. (I) Delineation of noteworthy metabolites in purine metabolism pathway. (J) Systematic decline of collective Z‐scores across stages.

Not only the diagnosis of CRC, but also the identification of the early versus late stages of CRC holds importance in clinical cares. To delve into CRC progression monitoring, we focused on a subset of CRC patients in our analyses, in which we have detailed staging information to investigate the alterations in metabolic profiles across varying tumor and lymph node (LN) stages. Figure 3A stratified these study subjects into groups without metastasis and with LN metastasis, subsequently subdividing them based on both T and N stages. Initially, a PLS‐DA model was constructed for with and without metastasis groups, as depicted in Figure 3B. However, the observed separation was inadequate, as evidenced by modeling parameters (*R* ^2^ = 0.26 and *Q* ^2^ = −0.09). Consequently, PLS‐DA models were also applied to compare solely T and N stages, as shown in Figure 3C,D. Although the stage‐based separations remained suboptimal, discernible trends were indicated by the directional changes as indicated by arrows in these figures. To validate these trends, TCGA transcriptomic data (Figure S1A,B) were utilized to construct PLS‐DA models by stages, as demonstrated in Figure S1C,D, of which the changing trends align with our metabolomic data‐based models. Furthermore, permutation tests with *p* > 0.05 for these aforementioned models suggested the necessity of a feature selection process to prevent overfitting. A volcano plot (Figure 3E) facilitated the selection of significantly altered metabolites in both with and without metastasis groups, revealing a notable increase in pyruvic acid and a decrease in phenol in LN metastasis cases. Upon examination of these compounds within subgroups (Figure 3F,G), a linear change by N stage, rather than T stage, was evident. This linear stage‐dependent change, observed in both PLS‐DA plots and selected compounds, prompted the selection of all significantly altered compounds by either T or N stages, as depicted in Figure 3H, Figure S2E. In total, 23 and 72 compounds were selected and visualized in heatmaps for T stage and N stage, respectively. Overall, there is an observed downward trend in compound levels as stages advanced, coupled with the stronger association of N stage with metabolic profile alterations, underscores the relevance of LN stage progression in CRC. Among the 23 T stage‐associated metabolites that have a general linear relationship with the stages, a principal pathway of influence, purine metabolism, was discerned. Noteworthy metabolites with significant z‐scores were delineated in Figure 3I, depicting a subtle declining trend across the stages. The collective z‐scores, as delineated in Figure 3J, further underscore a systematic decrease across the stages.

**FIGURE 4:**
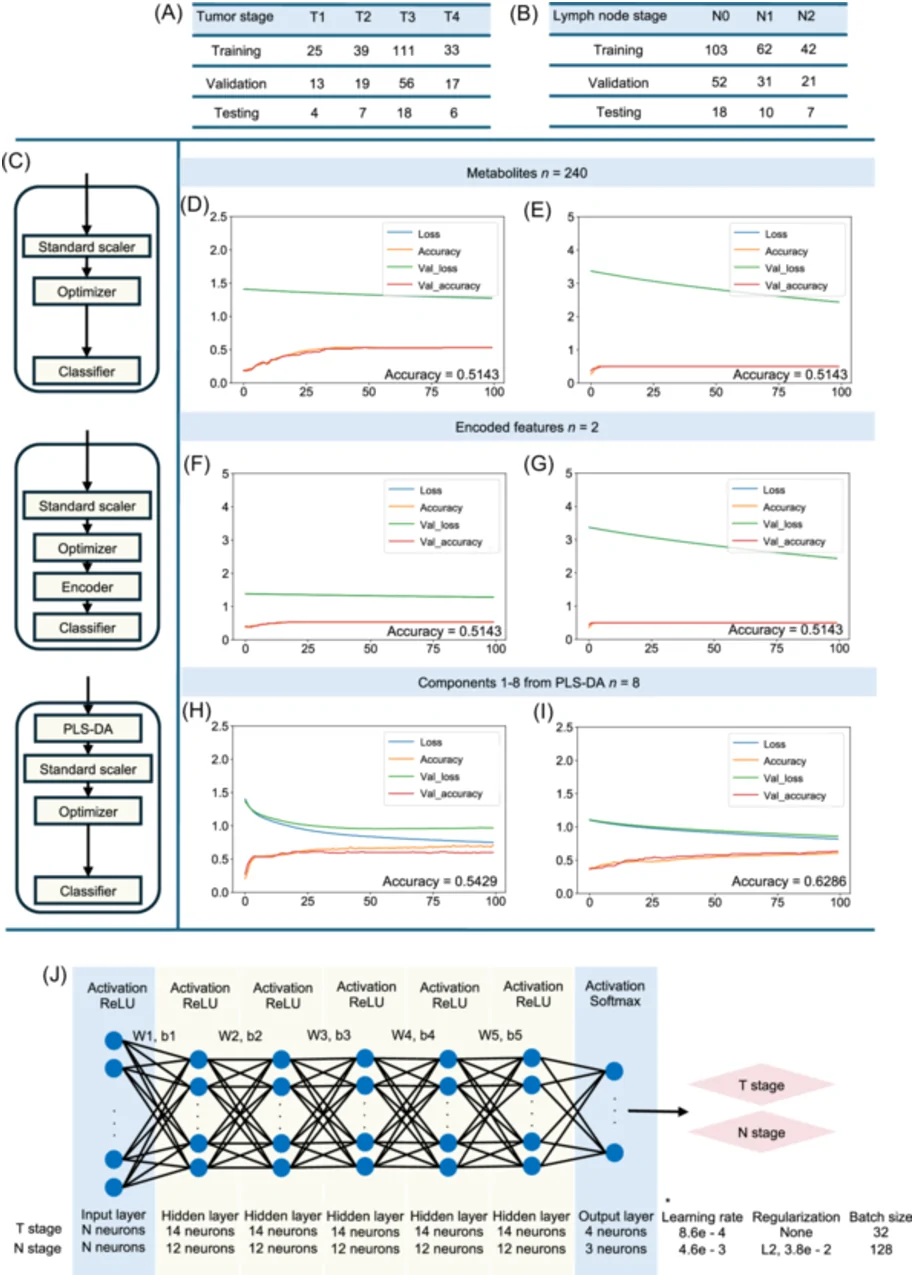
Stratification of colorectal cancer (CRC) subsets by tumor and lymph node stages, ANN workflow for stage classification, and loss/accuracy metrics for different models and components. (A) Stratification of CRC subset with detailed staging information by tumor (T) stage. (B) Stratification of CRC subset with detailed staging information by lymph node (N) stage. (C) ANN workflow for T and N stage classification. (D) Loss and accuracy metrics ‐ all metabolites (training set). (E) Loss and accuracy metrics ‐ all metabolites (validation set). (F) Loss and accuracy metrics ‐ autoencoding (2D) (training set). (G) Loss and accuracy metrics ‐ autoencoding (3D) (validation set). (H) Loss and accuracy metrics ‐ partial least squares discriminant analysis (PLS‐DA) components (training set). (I) Loss and accuracy metrics ‐ PLS‐DA components (validation set). (J) Hyperparameter optimization outcomes.

Given the suboptimal separation observed in the PLS‐DA models concerning T and N stages, along with indications of overfitting during CRC progression analysis, we turned to ANN‐based models to classify different T and N stages. As delineated in Figure 4A,B, all samples from the subset with detailed staging information were stratified according to T and N stages, respectively. Subsequently, the training and validation sets underwent processing through the three ANN workflows outlined in Figure 4C, spanning 100 epochs. The loss and accuracy metrics for both the training and validation sets across various data classifications are shown in Figure 4D–I, with the corresponding accuracy of the testing set indicated within the corresponding panels. Notably, when incorporating all 240 metabolites into the ANN classifier, decrease in loss (blue line) and increase in accuracy (orange line) of the training set were observed over epochs, while those for the validation set (green and red line) remained relatively stable (Figure 4D,E). Conversely, when employing autoencoding to compress all metabolites into 2 or 3‐dimensional spaces, the alignment of curves between the training and validation groups improved, indicating reduced overfitting (Figure 4F,G), alongside an increased accuracy for testing set (0.5143 for both T stage and N stage). Furthermore, PANDA pipeline, leveraging the eight components, yielded superior performance across the training, validation, and testing groups, with test set accuracies reaching 0.5429 for T‐stage classification and 0.6286 for N‐stage classification (Figure 4H,I). Notably, the optimized parameters for T stage and N stage differed, albeit both configurations encompassing five hidden layers, as delineated in Figure 4J, where other hyperparameter optimization outcomes were also documented. To demonstrate that such optimization is not a chance occurrence in metabolomic data, we subjected an equivalent classification of transcriptomic data to the same three pipelines. The results, as depicted in Figure S2, indicate that the PANDA pipeline significantly enhances the differentiation of T stage (accuracy = 0.8684) and N stage (accuracy = 0.6316).

### 2.4 Metabolomic and transcriptomic data integration highlighted the dysregulated TCA pathways in MLH1‐mutated CRC patients

**FIGURE 5:**
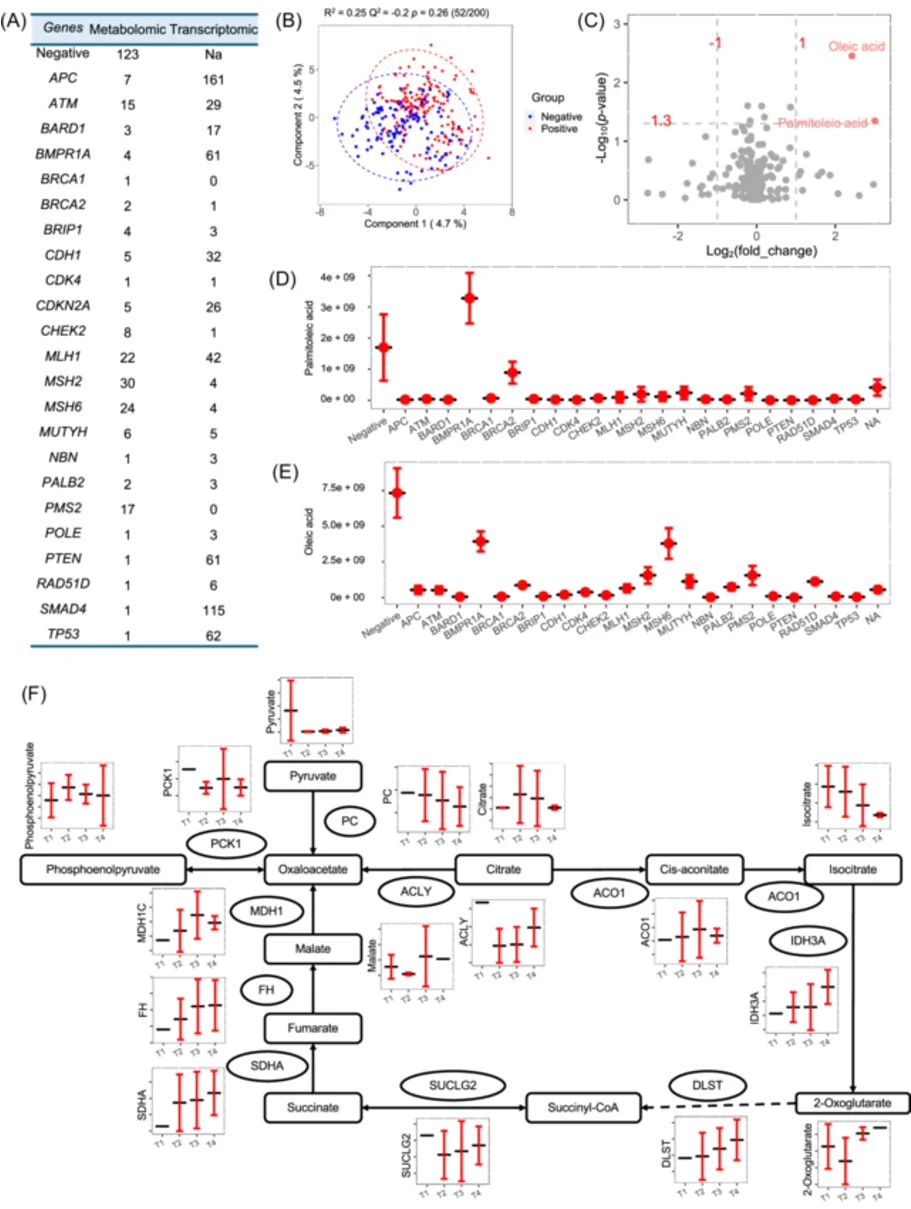
Stratification by gene mutation status, partial least squares discriminant analysis (PLS‐DA) model comparisons, metabolite alterations, and tricarboxylic acid (TCA) metabolism pathway analysis for MLH1 mutation status. (A) Stratification of CRC subset with detailed staging information by gene mutation status. (B) PLS‐DA model ‐ negative versus specific gene‐associated groups. (C) Significant metabolite alterations in negative cases. (D) The abundance of palmitoleic acid by specific gene‐associated groups. (E) The abundance of oleic acid by specific gene‐associated groups. (F) TCA metabolism pathway analysis of *MLH1* mutation status. CRC, colorectal cancer.

Additionally, our research includes specimens from individuals with inherited genetic mutations associated with conditions of CRC, such as Lynch syndrome. To elucidate the alterations in metabolic profiles that may connect progression and gene mutations, we conducted an analysis based on the gene mutation status of CRC cases with such information available. As our study also contains samples with genetic mutations that have been identified as heritable CRC risk factors. To elucidate the alterations in metabolic profiles that may connect progression and gene mutations, we conducted an analysis based on the gene mutation status of CRC cases with such information available. As illustrated in Figure 5A, samples from both our metabolomics study cohort and TCGA transcriptomic data were stratified into either negative or specific gene mutation‐associated groups. The inclusion of TCGA transcriptomic data facilitated pathway‐integrated analysis and validation of our findings from the metabolomics‐based analyses. A PLS‐DA model was employed to classify samples with or without genetic mutations, as depicted in Figure 5B, albeit with only a partial separation was achieved. Feature selection was subsequently performed, revealing significant decreases in oleic acid and palmitoleic acid levels in gene mutation‐associated positive cases (Figure 5C). Upon examination of these two compounds across all specific gene‐associated groups (Figure 5D,E), it was confirmed that they were significantly elevated in negative cases, with some exceptions noted in the *BMPR1A*, *BRCA2*, and *MSH6* groups. Given the limited overlap in sample amount (Figure 5A), *MLH1* was selected as our focus for pathway analysis. All significantly changed metabolites and mRNA data with linear trend were utilized as inputs for pathway analysis, and this integration revealed that the tricarboxylic acid (TCA) cycle is the most influenced pathway by CRC progression in this subgroup of patients (Figure 5F). Notably, within this pathway, the majority of mRNA exhibited an increasing trend, whereas the levels of metabolites displayed a decreasing trend by T stages. Similar trend of mRNA and metabolites change is also displayed in the pathway by N stages, as demonstrated in Figure S3.

## 3 DISCUSSION

In this study, we employed two distinct benchmark bioinformatic methods, PLS‐DA and ANN, along with their concatenated pipeline, PANDA, to analyze two different types of omics data. Our PANDA pipeline marks the first instance of integrating these two methodologies to generate large‐batch omics data for CRC occurrence prediction and progression monitoring. Our objective was to identify the alterations in metabolic profiles associated with CRC diagnosis and progression and further discover metabolic changes that can be used to stratify tumor stages and lymph node stages. Our long‐term goal is to either utilize these discovered biomarkers to enhance and support the current diagnostics by potentially adding these measurements in future clinical practices or generate machine learning‐supported biomarker groups that are to be utilized independently either as confirmatory tests or as screening tools for CRC diagnosis.

The highlight of the study lies in the development of our novel bioinformatic pipeline PANDA that allows simultaneous utilization of PLS‐DA and ANN, therefore offering a multi‐dimensional approach to data analysis. These two methods exhibit distinct advantages and applicability in feature selection and pattern recognition, thereby enhancing the comprehensiveness and reliability of the analysis. PLS‐DA possesses the capability for feature selection, identifying the most significant features for classification and differentiation among different groups [17]. However, PLS‐DA may not eliminate redundant data, potentially leading to overly complex models or unnecessary features. In contrast, ANN functions as a black‐box model [18]; although unable to conduct feature selection, it can achieve the removal of redundancy and improve data discrimination through weight adjustments. Additionally, ANN exhibits robust model fitting capabilities and nonlinear modeling, enabling better capture of complex patterns and associations within the data [19]. In our investigation, it was generally observed that ANN outperformed PLS‐DA in classification tasks pertaining to both diagnosis and progression analysis. This phenomenon is also substantiated by the fact that in the comparison between CRC and HC, only one hidden layer was incorporated. However, for the analysis of more intricate stages, such as those encountered in more complex scenarios, five hidden layers were introduced. Through cross‐validation, model robustness and reliability are strengthened, bolstering confidence in the consistency of results. Moreover, discrepancies in results between the two methods serve as cues for researchers to delve deeper into the data and models to ascertain genuine patterns and trends. Another notable aspect of this study entails the integration of metabolomic and transcriptomic data. While conventional multi‐omics analysis often involves correlation analysis [20], such approach cannot be applied in this context due to the disparate nature of the cohorts. Hence, within our investigation, we systematically apply identical methodologies, encompassing PLS‐DA and ANN, across diverse types of omics data. Subsequently, we integrate these disparate datasets into coherent pathways to consolidate the wealth of information therein. Furthermore, this integration process serves a dual purpose, as the interlacing of these datasets facilitates mutual validation, enhancing the robustness and reliability of our findings. We acknowledge that a limitation of this study is the absence of an independent external test set, as data was limited to two central sources (OCCPI and The Ohio State University Medical Center). This may affect the broader applicability of the model's high performance. Future work will incorporate external data to further validate its generalizability.

In the comparative analysis between CRC and HC cases, all prominently influenced pathways were upregulated (Figure 2J). This upregulation observed in the pathways align with previous studies, including arginine and proline metabolism [21, 22, 23], citrate cycle (TCA cycle) [24, 25], glycine, serine, and threonine metabolism [26, 27, 28], and purine metabolism [29, 30, 31] within the context of CRC. This can be attributed to underlying alterations in cellular energy metabolism and dysregulated biosynthesis and proliferation. The citrate cycle (TCA cycle) serves as a pivotal pathway for cellular energy production [32], while arginine and proline metabolism [21, 22], glycine, serine, and threonine metabolism [33, 34] also contribute significantly to energy metabolism and biosynthesis pathways. The observed upregulation of these pathways in CRC may signify a reprogramming of cellular energy metabolism, potentially favoring pathways conducive to tumor growth and proliferation. Furthermore, glycine, serine, and threonine metabolism play critical roles in the synthesis of nucleotides, proteins, and other essential biomolecules required for cellular growth, and proliferation [33, 35]. Similarly, purine metabolism is indispensable for nucleotide synthesis and DNA replication [36, 37]. The upregulation of these pathways in CRC may reflect alterations in biosynthetic processes and cellular proliferation dynamics, further contributing to the tumorigenic phenotype observed in CRC.

Meanwhile, the progression of CRC, as reflected in our metabolomics and transcriptomics analyses, is also intricately linked to the tumor (T) stage [38], indicating the degree of tumor invasion into the intestinal wall, and the lymph node (N) stage [39], reflecting the extent of cancer spread to nearby lymph nodes. Initially, we observed relatively linear changes in metabolites abundance by stages in metabolic profiles within PLS‐DA models; however, these patterns lacked clarity due to the overlap of PLS‐DA 95% CI areas. Therefore, ANN was employed to validate and extend our understanding of these trends to additional testing datasets. Notably, in cases of lymph node metastasis compared to those without metastasis, there was a significant increase in pyruvic acid levels and a decrease in phenol levels. These metabolic alterations are likely associated with the metastatic process. Pyruvic acid, a crucial metabolite in glycolysis [40], has been shown to be upregulated in cancer cells to support rapid proliferation and energy requirements, reflecting enhanced glycolytic activity in metastatic cells. Conversely, phenol, derived from dietary compounds and gut microbial metabolism, has exhibited decreased levels in lymph node metastasis cases, possibly indicating alterations in gut microbial composition [41] due to tumor progression in CRC.

The dysregulation of purine metabolism in CRC progression was discovered in our study when we stratified the patients and focused on the ones from T stages of CRC. As our results suggested a notable downregulation as tumors advanced through different stages. This is in contrast with the observed upregulation of purine metabolism when comparing CRC cases with HC. The observed upregulation of purine metabolism in CRC underscores the heightened metabolic activity requisite to sustain the rapid proliferation of cancer cells [42]. However, as CRC advances through various stages, including increasing T stages, the dynamics of purine metabolism undergo notable shifts. Dysregulated oncogenic signaling pathways, notably the Wnt/β‐catenin pathway, exert a critical influence on CRC progression and have the capacity to modulate metabolic pathways, including purine metabolism [30]. While the activation of these pathways may initially promote the upregulation of purine metabolism, their sustained activation may ultimately precipitate its downregulation in the advanced stages of CRC. Moreover, the tumor microenvironment, characterized by heightened hostility and nutrient deprivation as tumors progress, further exacerbates the downregulation of purine metabolism. This deprivation limits the availability of essential substrates [43] and cofactors necessary for purine metabolism [44], thereby contributing to its attenuated activity in advanced CRC stages.

## 4 CONCLUSION

In this comprehensive study, we developed and employed a novel machine‐learning pipeline, PANDA, to discover metabolic dysregulations derived from two different omics data types. Our primary aim was to elucidate alterations in metabolic profiles associated with CRC diagnosis and progression, stratified by tumor stages and lymphatic involvement. Our findings revealed significant upregulation in prominently influenced pathways when comparing CRC and HC cases, including arginine and proline metabolism, citrate cycle (TCA cycle), glycine, serine, and threonine metabolism, and purine metabolism, indicative of underlying alterations in cellular energy metabolism and dysregulated biosynthesis and proliferation pathways. Moreover, distinct metabolic changes associated with CRC progression, particularly in cases of lymph node metastasis, were observed, suggesting potential biomarkers for prognostic evaluation. Integration of metabolomic and transcriptomic data further strengthened our findings, facilitating results validation and enhancing the robustness of our conclusion. Overall, our study provides valuable insights into the metabolic dysregulation associated with CRC and underscores the potential of multi‐omics approaches in elucidating the intricate molecular mechanisms underlying cancer progression, which may allow the application of PANDA not only to CRC but also to other types of cancer studies in the future.

## 5 METHODS

### Chemicals and reagents

All the solvents used in this study, including LC/MS grade methanol, acetonitrile, water, ethanol, dimethyl sulfoxide, ammonium acetate, and acetic acid, were purchased from Fisher Scientific. Stable isotope‐labeled amino acid standards, ^13^C/^15^N‐AminoAMix20 were purchased from Cambridge Isotope Laboratories to be used as internal standards for biological samples. Metabolite standards were purchased from MetaSci.

### Sample collection

In total, three cohorts of samples originating from two distinct sources were gathered, with comprehensive characteristic details provided in Table 1. The first cohort (CRC 1) comprises 427 plasma samples obtained from The Ohio Colorectal Cancer Prevention Initiative (OCCPI) study (ClinicalTrials.gov identifier: NCT01850654), a prospective State of Ohio statewide study of universal screening for hereditary CRC [45]. Institutional review board approval was obtained by the individual hospitals, National Cancer Institute Community Oncology Research Programs participating in the study, or ceding review to the OSU Institutional Review Board (2012C0123). Additional plasma samples, which included the second cohort (CRC 2) of CRC patients (*n* = 199) and healthy control (HC) individuals (*n* = 402), were collected from The Ohio State University Medical Center Clinical Laboratory under IRB#2022C0138. HC individuals had to meet stringent exclusion criteria, which encompassed a range of medical conditions such as cardiac vascular disease (including, but not limited to, hypertension, myocardial infarction, valvular disease), diabetes (both type 1 and type 2), Crohn's disease, ulcerative colitis, hyperlipidemia, sepsis, shock, transgender individuals, thyroid disease, cancer patients undergoing chemotherapy, and pregnancy (female). All samples were collected after overnight fasting and aliquoted and stored at −80°C freezer until analyses.

**TABLE 1:** The characteristic information of local colorectal cancer (CRC) patients and healthy control (HC) individuals.

Cohort	CRC 1	CRC 2	HC	Sum
Sum	427	199	529	1155
Sex				
Female	218	89	274	581
Male	209	110	255	574
Age (years)				
10–20	0	1	18	19
20–30	5	0	166	171
30–40	45	8	84	137
40–50	143	28	13	184
50–60	87	64	109	260
60–70	70	53	114	237
70–80	59	37	18	114
80–90	17	7	7	31
90–100	1	1	0	2
Age average in years (standard deviation)	54.4 (13.4)	59.9 (12.2)	50.1 (15.3)	
Race/ethnicity				
Asian	2	4	9	15
Black	20	21	37	78
Caucasian	399	169	183	751
Hispanic	2	3	7	12
Multiracial	1	1	0	2
Unknown	3	1	1	5
BMI (Kg/m^2^)				
<18.5	4	80	407	4
18.5–24.9	74	0	0	74
25–29.9	103	1	1	105
>30	86	118	121	325
Not available	160	0	0	647

Abbreviations: CRC, colorectal cancer; BMI, body mass index; HC, healthy control.

#### 5.1 Standard preparation and metabolite extraction

To prepare the metabolite standards for the study, each standard was dissolved in the most suitable solvent (water, ethanol, or dimethyl sulfoxide) based on their solubility. Stock solutions of 10 mM concentration were prepared for each standard. A gradient of standards ranging from 10 μM to 1 mM was generated by diluting the stock solutions. These gradients were used for direct infusion analysis. Additionally, mixes of each standard at a concentration of 10 μM were prepared to determine the retention times.

For polar metabolites extraction, 50 µL plasma from each sample was aliquoted and mixed with ice‐cold methanol and ^13^C^15^N‐labeled internal standard. After vortex, samples were stored in −20°C freezer for 20 min to precipitate protein. After centrifugation at 14,000 rpm for 20 min, the 150 µL supernatant was carefully transferred in clean LC‐MS vials and loaded onto temperature controlled (4°C) sample tray in Vanquish UHPLC System (Thermo Fisher).

### Metabolomic data collection

Randomization was rigorously implemented for the entirety of plasma sample collection, resulting in a total of 11 analytical batches. To account for instrument variability over prolonged data collection periods, a human serum quality control (HSQC) sample and a blank sample (methanol) were intercalated between every 10 consecutive samples. Within each batch, five injections of pooled quality control (PQC) were conducted, with four injections designated for targeted compound scanning and one for full scan with MS^2^ analysis. This comprehensive approach facilitated both target detection and annotation of untargeted compounds based on reference databases, ensuring robustness and accuracy in data interpretation. Both positive and negative ionization mode were performed for data collection of each batch.

All the samples were analyzed by the Thermo Vanquish UHPLC system coupled with a Q‐Exactive Orbitrap mass spectrometer equipped with a heated electro‐spray ionization probe (Thermo Fisher) as well as a XBridge BEH Amide XP column (130 Å, 2.5 µm, 2.1 mm × 150 mm, 2.5 μm particle size; Waters Corporation, Milford, MA). For the chromatographic analysis, mobile phase A comprised a mixture of 5 mM ammonium acetate in acetonitrile/water (10:90, v/v) with 0.1% acetic acid, while mobile phase B was composed of 5 mM ammonium acetate in acetonitrile/water (90:10, v/v) with 0.1% acetic acid. A linear gradient elution program was implemented, initiating with 70% B and gradually decreasing to 30% B within 5 min. Subsequently, the mobile‐phase composition was sustained at 30% B for 4 min, after which it was reverted to 70% B within 2 min and maintained for an additional 2 min. The entire run duration lasted 13 min. The flow rate was set to 0.3 mL/min, and the column temperature was maintained at 40°C.

### Metabolomic data preprocessing

Raw mass spectroscopy data for biological samples and targeted list PQCs were converted to a universal format (.mzXL) by Proteo Wizard for analysis for spectral peak extraction using the XCMS R package. Then PQCs were annotated by comparing with our commercial standards. PQC samples with the full scan and MS/MS spectra were analyzed by Compound Discoverer to extract peak information and annotated compounds using Mzcloud database (https://www.mzcloud.org). Metabolite alignment from PQC to other samples uses in‐house R code with the criteria of retention time (rt) < 120 s and mass‐to‐charge ratio (m/z) < 10 parts per million (ppm). Only MS/MS level annotation were kept for further analysis.

The coefficient of variation (CV) was calculated for the averages of HSQC samples within a batch. Peaks meeting the criteria of CV less than 0.3 and an average intensity greater than 1e5 were kept. Normalization strategies were evaluated, considering batch‐wise, per‐ten‐sample, or combined approaches. Ultimately, batch‐wise normalization was selected as the preferred method. Log transformation and auto scaling were employed for feature normalization, while mean values for each metabolite were utilized for missing value imputation. Eventually, 240 metabolites were retained for further analysis (Table S1). PQCs were employed to validate normalization. Following normalization (Figure S4B), the distribution levels of all metabolites across 11 batches were notably more consistent compared to before (Figure S4A).

### Transcriptomic data access and preprocessing

The normalized colon adenocarcinoma mRNAseq data and clinical data were obtained from the Broad Institute (https://gdac.broadinstitute.org). The Cancer Genome Atlas (TCGA) initiative, accessible for global research, aggregates diverse genomic, epigenomic, transcriptomic, and proteomic data to advance cancer diagnosis, treatment, and prevention. Using R Studio (version 4.2.2), gene expression data was integrated with clinical data and samples were categorized into CRC and HC groups, as well as different T and N stages. For the gene mutation‐associated subset, we identified significantly decreased expression of the *MLH1* gene in patients, with a fold change < 0.5 and an FDR *p*‐value < 0.05, thereby retaining it as a subset associated with *MLH1* gene mutation.

### Statistical analysis

PLS‐DA was applied to both metabolomics and transcriptomic datasets to distinguish between groups and pinpoint significant features specific to each data type. Separately, ANNs were trained on both metabolomics and transcriptomic data to verify whether the distinctions identified by PLS‐DA could be generalized and to explore latent patterns in each data set independently.

### Integrated pathway analysis

Significantly different metabolites and genes were selected and compared to KEGG library [46] together for integrated gene‐metabolite interaction pathway analysis. By examining these interactions within shared metabolic pathways, the metabolomics and transcriptomics datasets were integrated, allowing for a comprehensive view of pathway‐level changes. The z‐score was used to quantify the deviation of observed pathway activity from what would be expected by chance, with higher scores indicating pathway enrichment and lower scores indicating depletion, thus providing a measure of the biological significance of the pathway.

## AUTHOR CONTRIBUTIONS

**Rui Xu**: Conceptualization, data curation; formal analysis, investigation; writing–original draft. **Hyein Jung**, **Fouad Choueiry**, **Shiqi Zhang**, **Rachel Pearlman**, **Heather Hampel**, and **Ning Jin**: Investigation. **Jieli Li** and **Jiangjiang Zhu**: Supervision, writing–review & editing. All authors have read the final manuscript and approved it for publication.

## CONFLICT OF INTEREST STATEMENT

The authors declare no conflicts of interest.

## ETHICS STATEMENT

Institutional review board approval was obtained by the individual hospitals, National Cancer Institute Community Oncology Research Programs participating in the study, or ceding review to the OSU Institutional Review Board (No. 2012C0123) for the OCCPI samples. The ethics application (No. 2022C0138) was approved by The Ohio State University Medical Center Clinical Laboratory.

## Supporting information

The online version contains supplementary figures and tables available.

Figure S1. TCGA transcriptomic data stratified by T stage, N stage, and PLS‐DA model comparisons.
Figure S2. CRC transcriptomic data stratification, ANN workflow, and loss/accuracy metrics.
Figure S3. TCA metabolism pathway analysis of N stage classification (Transcriptomic Data).
Figure S4. Distribution of metabolite intensities in pooled QC samples before and after normalization.

Table S1. Metabolites annotated from both targeted method and untargeted method.

## Data Availability

The mass spectra data has been deposited to MassIVE database (https://massive.ucsd.edu/ProteoSAFe/static/massive.jsp) with access # MSV000096806. The code of our analysis algorithm and a small set of demo data have been made available in GitHub with the following link: https://github.com/Xrrrr98784/PANDA-workflow. Supplementary materials (figures, tables, graphical abstract, slides, videos, Chinese translated version, and update materials) may be found in the online DOI or iMeta Science http://www.imeta.science/imetaomics/.
