# Supplementary material for: Novel machine‐learning bioinformatics reveal distinct metabolic alterations for enhanced colorectal cancer diagnosis and monitoring
Source: IMetaOmics. 2025 Mar 3;2(2):e70003. doi: 10.1002/imo2.70003 (PMC12806213; doi:10.1002/imo2.70003)
Supplement: Supplementary file 1 — Figure S1. TCGA transcriptomic data stratified by T stage, N stage, and PLS‐DA model comparisons. Figure S2. CRC transcriptomic data stratification, ANN workflow, and loss/accuracy metrics. Figure S3. TCA metabolism pathway analysis of N stage classification (Transcriptomic Data). Figure S4. Distribution of metabolite intensities in pooled QC samples before and after normalization. [file IMO2-2-e70003-s002.docx]

# **Supplementary information to**

# **Novel machine-learning bioinformatics reveal distinct metabolic alterations for enhanced colorectal cancer diagnosis and monitoring**

**Running title:** Machine learning identifies metabolic shifts in colorectal cancer

Rui Xu^1, 2^, Hyein Jung^3^, Fouad Choueiry^1, 2^, Shiqi Zhang^1, 2^, Rachel Pearlman^2, 4^, Heather Hampel^5, 6^, Ning Jin^2^, Jieli Li^7^*, Jiangjiang Zhu ^1,2^*

^1^Human Nutrition Program, Department of Human Sciences, The Ohio State University, Columbus, Ohio 43210

^2^Comprehensive Cancer Center, The Ohio State University Wexner Medical Center, Columbus, Ohio 43210

^3^Department of Chemistry and Biochemistry, The Ohio State University, Columbus, Ohio 43210

^4^Department of Internal Medicine, The Ohio State University, Ohio 43210

^5^Department of Medical Oncology & Therapeutics Research, City of Hope, CA 91010

^6^Division of Clinical Cancer Genomics, Beckman Research Institute, City of Hope, CA 91010

^7^Department of Pathology, The Ohio State University, Columbus, OH 43210, USA

*Correspondence: [jieli.li@osumc.edu](mailto:jieli.li@osumc.edu) (Jieli Li, M.D., Ph.D.), zhu.2484@osu.edu (zhu.2484@osu.edu)

**Figure S1 TCGA transcriptomic data stratified by T stage, N stage, and PLS-DA model comparisons.** (A) TCGA transcriptomic data stratified by T stage. (B) TCGA transcriptomic data stratified by N stage. (C) PLS-DA model of tumor stage comparison (transcriptomic data). (D) PLS-DA model of lymph node stage comparison (transcriptomic data). (E) Selected altered compounds by tumor and lymph node (LN) stages (transcriptomic data).

**Figure S2 CRC transcriptomic data stratification, ANN workflow, and loss/accuracy metrics.** (A) Stratification of CRC subset of transcriptomic data with detailed staging information by tumor (T) stage. (B) Stratification of CRC subset of transcriptomic data with detailed staging information by lymph node (N) stage. (C) ANN workflow for T and N stage classification. (D) Loss and accuracy metrics - all mRNAs (training set). (E) Loss and accuracy metrics - all mRNAs (validation set). (F) Loss and accuracy metrics - autoencoding (2D) (training set). (G) Loss and accuracy metrics - autoencoding (3D) (validation set). (H) Loss and accuracy metrics - PLS-DA components (training set). (I) Loss and accuracy metrics - PLS-DA components (validation set). (J) Hyperparameter optimization outcomes.

**
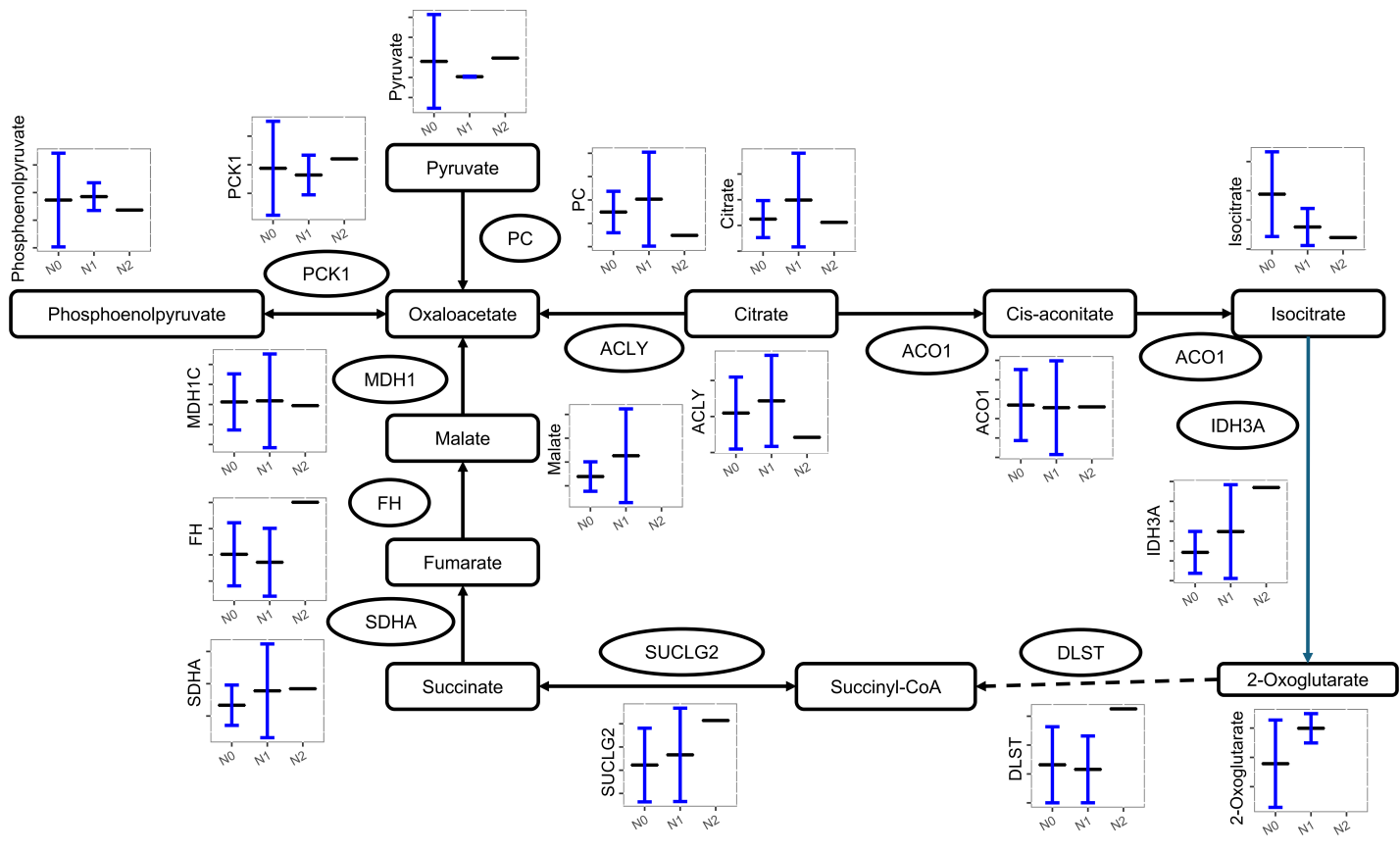
**

**Figure S3 TCA metabolism pathway analysis of N stage classification (transcriptomic data).**

**Figure S4 Distribution of metabolite intensities in pooled QC samples before and after normalization.** (A) The normalized intensity distribution of metabolites in pooled QC samples before normalization. (B) The normalized intensity distribution of metabolites in pooled QC samples after normalization.
